# The NLRP3 inflammasome pathway contributes to chronic inflammation in experimental autoimmune uveitis

**DOI:** 10.1002/ame2.70011

**Published:** 2025-03-29

**Authors:** Avik Shome, Ilva D. Rupenthal, Rachael L. Niederer, Odunayo O. Mugisho

**Affiliations:** ^1^ Buchanan Ocular Therapeutics Unit, Department of Ophthalmology New Zealand National Eye Centre, University of Auckland Auckland New Zealand; ^2^ Department of Ophthalmology New Zealand National Eye Centre, University of Auckland Auckland New Zealand; ^3^ Department of Ophthalmology Te Whatu Ora Te Toka Tumai Auckland New Zealand

**Keywords:** experimental autoimmune uveitis, inflammation, nucleotide‐binding oligomerization domain and leucine‐rich repeat receptor‐3 (NLRP3) inflammasome, uveitis

## Abstract

**Background:**

Noninfectious uveitis, a chronic ocular inflammatory disease, is characterized by the activation of immune cells in the eye, with most studies focusing on the role of the adaptive immune system in the disease. However, limited data exist on the potential contribution of the innate immune system, specifically the nucleotide‐binding oligomerization domain and leucine‐rich repeat receptor‐3 (NLRP3) inflammasome pathway. This pathway has previously been identified as a driver of inflammation in several low‐grade, progressive inflammatory eye diseases such as diabetic retinopathy. The aim of this study was to determine whether the NLRP3 inflammasome pathway plays a role in the pathogenesis and chronicity of experimental autoimmune uveitis (EAU).

**Methods:**

EAU was induced in C57BL/6J mice via intraperitoneal pertussis toxin and subcutaneous interphotoreceptor retinoid‐binding protein injections. After 12 weeks, eyes were enucleated, and whole eye sections were assessed for inflammasome, macrophage, and microglial markers in the retina, ciliary body, and cornea using immunohistochemistry.

**Results:**

Our study confirmed higher NLRP3 inflammasome activation (increased expression of NLRP3 and cleaved caspase 1 labeling) in EAU mouse retinas compared to controls. This correlated with increased astrogliosis and microglial activation throughout the eye. Migratory innate and adaptive peripheral immune cells (macrophages and leukocytes) were also found within the retina and ciliary body of EAU mice. Connexin43 proteins, which form hexameric hemichannels that can release adenosine triphosphate (ATP), an upstream inflammasome priming signal, were also found upregulated in the retina and cornea of EAU mice.

**Conclusion:**

Overall, our findings support the idea that in the EAU model there is active inflammation, even 12 weeks post induction, and that it can be correlated to inflammasome activation. This contributes to the pathogenesis and chronicity of noninfectious uveitis, and our results emphasize that targeting the inflammasome pathway could be efficacious for noninfectious uveitis treatment.

## Graphical abstract

The present study investigates the role of the NLRP3 inflammasome in the chronicity of ocular inflammation in uveitis using the experimental autoimmune uveitis (EAU) model. Whole eyes collected from EAU mice, 12 weeks post induction, were sectioned and subjected to immunohistochemistry. The confocal images from these sections were then assessed for the expression of general inflammation and inflammasome activity markers, comparing EAU to control mice.

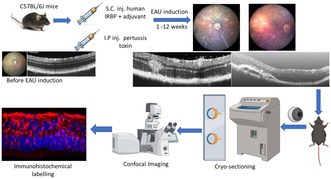

## 1 INTRODUCTION

The innate immune system forms the first line of defense against pathogens and is largely responsible for acute inflammatory response in pathogenic infections.^1^ This inflammatory response can lead to intraocular inflammation known as infectious uveitis that is resolved when the underlying infection is treated.^2^ In contrast, manifestation of noninfectious uveitis is largely associated with the development of systemic (e.g., juvenile idiopathic arteritis) or ocular (e.g., birdshot chorioretinopathy) autoimmune diseases due to a flawed adaptive immune system that is responsible for the associated chronic inflammation, necessitating prolonged treatment with corticosteroids.^3^ The T‐ and B‐cell receptors of the adaptive immune system are responsible for the production of pathogenic autoreactive CD4^+^ and CD8^+^ lymphocytes (e.g., T‐helper [Th]1, Th2, and Th17 cells) with self‐antigen‐recognizing receptors.^4^, ^5^, ^6^ These adaptive immune cells are thought to be the primary initiators of autoimmune diseases, with Th17 subset being predominantly responsible for the development of chronic inflammation in noninfectious uveitis.^7^ The differentiation of naive T cells into Th17 cells is regulated by pro‐inflammatory cytokines such as interleukin (IL)‐6, IL‐1β, and transforming growth factor‐β.^8^ Th17 cells primarily produce IL‐17 (IL‐17A and IL‐17F) that facilitates neutrophil, macrophage, and leukocyte infiltration into the eye as well as release of pro‐inflammatory cytokines such as IL‐6 and IL‐1β from immune and nonimmune cells. These cytokines form a self‐perpetuating inflammatory feedback loop resulting in chronic inflammation.^9^ However, the eye exists in a state of “immune privilege” due to the presence of the blood retinal barrier (BRB) and blood aqueous barrier with only a limited number of resident innate and adaptive immune cells.^10^, ^11^ It is still unclear how the immune cells ultimately infiltrate the eye during ocular inflammation in noninfectious uveitis.

The inflammasome complex is an integral part of the innate immune system. It contains nucleotide‐binding oligomerization domain (NOD) and leucine‐rich repeat (NOD‐like) receptors (NLR) attached to a protein scaffold (NLRP), apoptosis‐associated speck‐like protein containing caspase activation and recruitment domain adaptor and the cysteine protease caspase‐1.^12^ The assembly of the inflammasome complex is induced by pathogen‐associated molecular patterns or recognition of damage‐associated molecular patterns and sometimes, in the absence of pathogens, via a priming signal such as a mechanical force on the cell or excessive cytokine production as seen in an autoimmune disease. This priming signal is then followed by an activation signal such as recognition of adenosine triphosphate (ATP) by P2X7 receptors as well as Ca^2+^ and K^+^ fluxes (secondary signal). This then results in the assembly of the complex followed by autolytic cleavage of caspase‐1 (CC1 [cleaved caspase 1]), resulting in the maturation and release of pro‐inflammatory cytokines (IL‐1β and IL‐18), which triggers inflammation that is “sterile” (without pathogens) in nature.^12^, ^13^ Upstream of inflammasome activation is the process of pathologic connexin43 hemichannel opening that is responsible for the release of ATP into the extracellular space, providing the secondary signal.^14^ Most cells (endothelial, epithelial, and resident immune cells such as microglia) in the eye express NLRs and contain the components for assembly of NLR protein complexes, especially the nucleotide‐binding oligomerization domain and leucine‐rich repeat receptor‐3 (NLRP3), and can contribute to the production of pro‐inflammatory cytokines such as IL‐1β and IL‐18.^15^ This combined with the fact that IL‐1β regulates the proliferation and survival of Th17 cells as well as differentiation of naive T cells into the Th17 subset^16^ highlights a possible interaction of the innate and adaptive immune systems in uveitis.

Experimental autoimmune uveitis (EAU) rodent models have been used extensively to study various aspects of uveitis, including the role of pro‐inflammatory cytokines (e.g., IL‐1β),^17^ Th17 cells,^6^, ^18^ and NLRP3 activation^19^ in the development and proliferation of ocular inflammation in uveitis. In addition, the NLRP3 inflammasome plays a crucial part in other ocular inflammatory diseases, including glaucoma,^20^ age‐related macular degeneration (AMD),^21^ and diabetic retinopathy.^22^ In uveitis, several studies have reported inflammation peaks 21–28 days post EAU induction,^23^, ^24^, ^25^ with active inflammation resolving thereafter. However, we previously found that inflammation proceeds to 12 weeks after manifestation with inflammatory signs, especially those seen in optical coherence tomography (OCT) images, continuing to worsen over time.^26^ This study investigated whether the chronicity of uveitis could be governed in some part by the inflammasome pathway and, if so, how this might relate to other well‐known inflammatory markers, generally measured at 28 days post EAU induction. Cross sections of eye globes from EAU and control mice (12 weeks post immunization) were subjected to immunohistochemistry to measure general inflammation (glial fibrillary acidic protein [GFAP] and ionized calcium‐binding adapter molecule 1 [Iba‐1]), NLRP3 inflammasome activation (NLRP3 and CC1), and the upstream gap junction protein (connexin43) within the retina, ciliary body, and cornea. In addition, markers for Th17 cells (IL‐17A), leukocytes (CD45), and macrophages (CD68) were used to investigate the infiltration of innate and adaptive immune cells from the periphery.

## 2 MATERIALS AND METHODS

### 2.1 EAU induction and tissue collection

EAU was induced in 6‐ to 8‐week‐old female C57BL/6J mice (*n* = 6) by subcutaneous injection of 400 μg of human interphotoreceptor retinoid‐binding protein peptide 1–20 (GPTHLFQPSLVLDMAKVLLD, China Peptides, Shanghai, China) in complete Freund's adjuvant with a concurrent intraperitoneal injection of 1.5 μg of pertussis toxin (Enzo Life Sciences, Farmingdale, NY, USA) in 50 μL of saline as previously described.^26^ Control mice (*n* = 4) received no subcutaneous or intraperitoneal injections. After 12 weeks, mice were killed using carbon dioxide gas (3 L/min), and then cervical dislocation was performed. Eyes were enucleated, and globes were fixed in 4% paraformaldehyde aqueous solution (ProSci Tech, Kirwan, QLD, Australia) for 1 h. Eye globes were then washed in 0.1 M phosphate buffered saline (PBS) solution and passed through a sucrose gradient. Eyes were then embedded in an optimal cutting temperature medium (Leica Biosystems Richmond Inc., Richmond, IL, USA), sectioned (14 μm) at −20°C using a cryostat (CryoStar Nx50, Thermo Scientific, Waltham, MA, USA), and mounted on Superfrost glass slides (Electron Microscopy Sciences, Hatfield, PA, USA). All procedures were carried out in accordance with local legislations approved by the University of Auckland Animal Ethics Committee (AEC2882 approved on November 9, 2020) and in accordance with the Association for Research in Vision and Ophthalmology resolution and the animal research: reporting of in vivo experiments (ARRIVE) guidelines for use of animals in research.

### 2.2 Immunohistochemistry

Sections were washed thrice for 5 min each time in PBS and then blocked for 1 h with 10% goat or horse serum in 0.1% Triton X‐100 in PBS. The sections were then incubated overnight with primary antibodies at 4°C (Table 1). After overnight incubation, the sections were washed thrice for 5 min each time in PBS to remove any excess antibody and were then incubated with the corresponding secondary antibody (Table 1) as well as the cell nuclei stain (4′,6‐diamidino‐2‐phenylindole) DAPI (0.5 μg/mL, D9542, Sigma‐Aldrich, St. Louis, MO, USA) for 2 h at room temperature. The sections were washed again thrice for 5 min each time in PBS before being mounted with an antifade medium (Citifluor, Electron Microscopy Sciences).

**TABLE 1:** Sets of primary and secondary antibodies used in this study.

Primary antibody	Role	Dilution	Secondary antibody	Dilution	Tissues with positive staining
**GFAP‐Cy3** (C2905, Sigma‐Aldrich, St. Louis, MO, USA)	Müller cell and astrocyte marker	1:1000	**N/A**	N/A	Retina
**Iba‐1** (ab178846‐Abcam plc, Cambridge, UK)	Microglia marker	1:2000	**Goat α rabbit Alexa Fluor 488** (A11034, Invitrogen, Waltham, MA, USA)	1:500	Retina Ciliary body Cornea
**CD68** (137 002, BioLegend Inc., San Diego, CA, USA)	Macrophage marker	1:500	**Goat α rat Alexa Fluor 488** (A11006, Molecular Probes, Eugene, OR, USA)	1:500	Retina
**CD45** (103 102, BioLegend Inc.)	Leukocyte marker	1:500	**Goat α rat Alexa Fluor 488** (A11006, Molecular Probes)	1:500	Retina Ciliary body
**IL‐17A** (ab79056, Abcam plc)	Th17 cell marker	1:200	**Goat α rabbit CY3** (111‐165‐003, Jackson ImmunoResearch Labs, West Grove, PA, USA)	1:500	Retina Ciliary body
**NLRP3** (ab4207, Abcam plc)	Inflammasome marker	1:500	**Donkey α goat Cy3** (705‐165‐147, Jackson ImmunoResearch Labs)	1:500	Retina Ciliary body Cornea
**CC1** (PA5‐38099, Invitrogen)	Inflammasome activation marker	1:50	**Goat α rabbit Alexa Fluor 488** (A11034, Invitrogen)	1:500	Retina Ciliary body Cornea
**Connexin43** (C6219, Sigma‐Aldrich)	Upstream inflammasome marker	1:2000	**Goat α rabbit Cy3** (111‐165‐003, Jackson ImmunoResearch Labs)	1:500	Retina Ciliary body Cornea

*Note:* Bold represent the names of the primary and secondary antibodies used in the study. Showing these words in bold is simply to allow the names to stand out compared to the cat Numbers and company information in brackets. Abbreviations: CC1, cleaved caspase 1; GFAP, glial fibrillary acidic protein; Iba‐1, ionized calcium‐binding adapter molecule 1; IL, interleukin.

### 2.3 Imaging and quantification

All images were obtained using an Olympus FV1000 confocal laser scanning microscope (Olympus, Tokyo, Japan). For each marker, six images were taken per eye for the retina and cornea, whereas four images were taken for the ciliary body. The researcher was masked during immunofluorescence imaging to reduce bias. Each image was quantified using ImageJ software version 1.46r (National Institutes of Health, Bethesda, MD, USA) by a masked researcher to avoid bias. To quantify IL‐17A, combined IL‐17A and DAPI images were initially used to select the area of the retina, ciliary body, and cornea using the freehand drawing tool. The IL‐17A^+^‐only channel was then converted into an 8‐bit image. Using images with the highest labeling intensity, a threshold was set and applied to all images to reduce any background. The percentage area covered by IL‐17A^+^ labeling and the mean fluorescence intensity (MFI) were quantified using the measure tool. Similarly, the percentage area covered by CD45^+^ and CD68^+^ labeling was quantified in the retina, ciliary body, and cornea. GFAP, Iba‐1, NLRP3, CC1, and connexin43 were quantified as explained previously.^27^ NLRP3, CC1, and connexin43 were also quantified in individual layers of the retina, namely the ganglion cell layer (GCL), inner plexiform layer (IPL), inner nuclear layer (INL), outer plexiform layer (OPL), and outer nuclear layer (ONL). Moreover, the expression of markers in specified regions of the ciliary body (ciliary processes [P], outer pigmented epithelium [OPE], inner nonpigmented epithelium [INPE]) and the cornea (corneal epithelium [Epi], stroma, corneal endothelium [Endo]) was observed.

### 2.4 Statistical analysis

All statistical analyses were conducted using MS Excel 2021 and GraphPad Prism software, version 9 (GraphPad Software, San Diego, CA, USA). Data was presented as an arithmetic mean + standard error of the mean. Statistically significant difference (*p* ≤ 0.05) between the groups was determined using Student's unpaired *t*‐test or two‐way analysis of variance (ANOVA) with Sidak's multiple comparisons post hoc test. Simple linear regression was calculated between the expression of IL‐17A, NLRP3, CC1, and connexin43. The specific test used is mentioned in the figure legends.

## 3 RESULTS

### 3.1 GFAP was upregulated in the retina, and Iba‐1^+^ cells increased in the retina, ciliary body, and cornea in EAU mice

**FIGURE 1:**
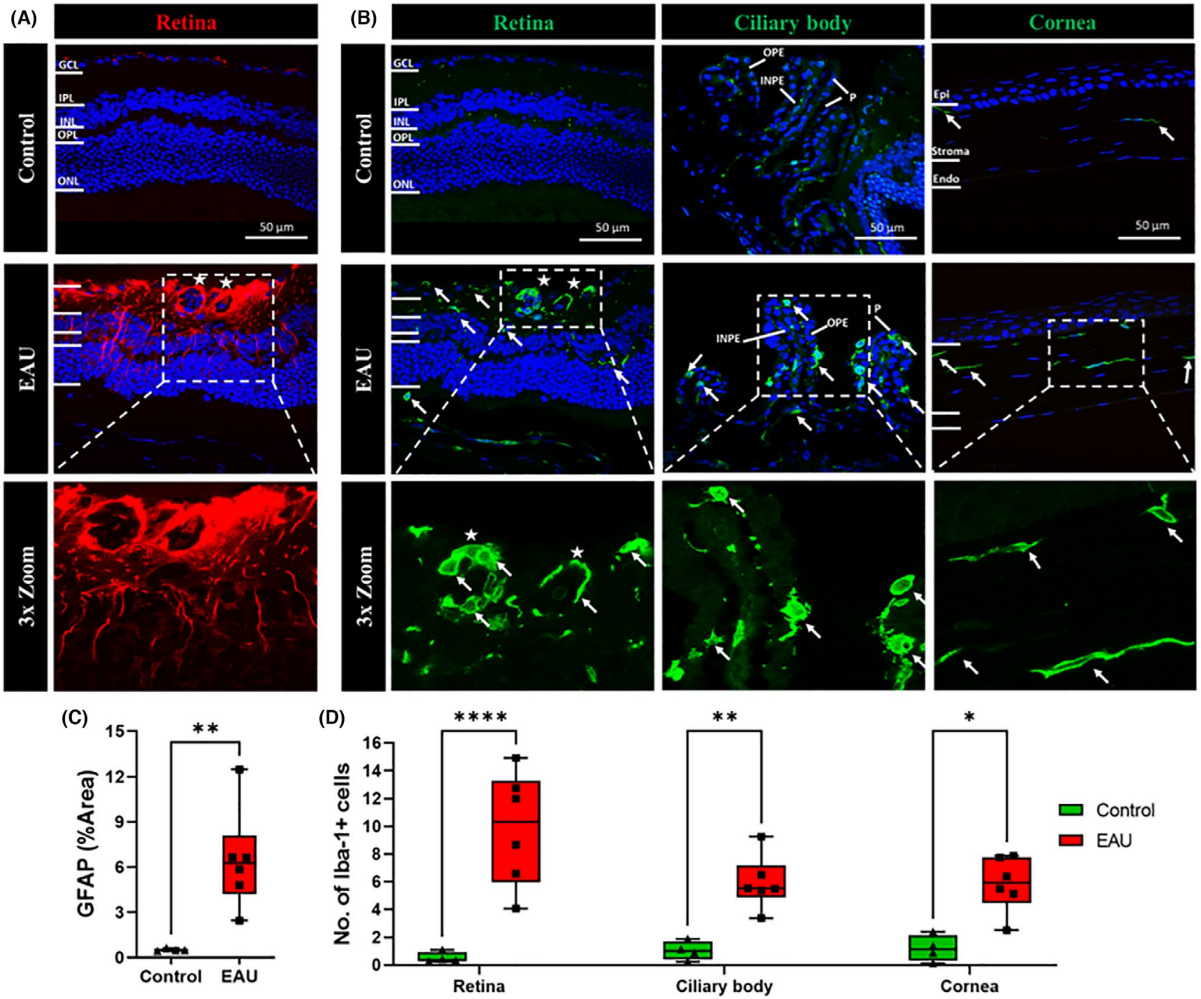
Expression of inflammatory markers (GFAP [glial fibrillary acidic protein] and Iba‐1 [ionized calcium‐binding adapter molecule 1]) in the retina, ciliary body, and cornea in EAU (experimental autoimmune uveitis) mice (*n* = 6) compared to controls (*n* = 4). (A) Expression of GFAP (red) was upregulated throughout all layers of the retina with high expression around granulomas (white stars). The intrusion of astrocytes from the GCL (ganglion cell layer) into the IPL (inner plexiform layer), INL (inner nuclear layer), ONL (outer nuclear layer), and OPL (outer plexiform layer) was clearly observed in EAU mice. (B) Iba‐1 (green) expression was upregulated in the GCL, IPL, OPL, and ONL (white arrows) as well as around clumps of cells present in the GCL (white stars) of the retina, OPE (outer pigmented epithelium), and INPE (inner nonpigmented epithelium) (white arrows) of the ciliary body and in the corneal stroma (white arrows) of EAU mice compared to controls. (C) Significant differences in GFAP expression in the retina between the EAU and control groups were calculated using Student's *t*‐test. The total area of the retina expressing GFAP was significantly higher (**p = 0.0070) in EAU mice compared to controls. (D) Significant differences in Iba‐1^+^ cells in the retina, ciliary body, and cornea between the EAU and control groups were calculated using two‐way ANOVA (analysis of variance) with Sidak's multiple comparisons post hoc test. EAU mice had a significantly higher count of Iba‐1^+^ cells in the retina (****p < 0.0001), ciliary body (**p = 0.0090), and cornea (*p = 0.0100).

Inflammatory stress in the retina, ciliary body, and cornea was quantified by measuring the expression of GFAP and Iba‐1. Results showed that GFAP labeling extended from the GCL to the ONL in EAU mice but was restricted to the GCL only in control mice (Figure 1A). When GFAP labeling was quantified (Figure 1C), the results showed that the protein was significantly upregulated in EAU mice (6.447% ± 1.358% vs. 0.495% ± 0.043%, *p* = 0.0070) compared to controls. There was no GFAP expression in the ciliary body or cornea.

There was an increase in the number of Iba‐1^+^ cells in the GCL, IPL, OPL, and ONL in EAU mice compared to controls (Figure 1B). Increased numbers of Iba‐1^+^ cells were also observed in the OPE and INPE of the ciliary processes (villi‐like projections; P) comprising the ciliary body and in the corneal stroma in EAU compared to control mice. When Iba‐1^+^ cells were counted, a significantly increased number of cells were observed in the retina (9.8 ± 1.6 vs. 0.5 ± 0.1, *p* < 0.0001), ciliary body (5.9 ± 0.7 vs. 1.0 ± 0.3, *p* = 0.0090), and cornea (5.8 ± 0.8 vs. 1.2 ± 0.5, *p* = 0.0100) of EAU mice compared to respective tissues in control mice (Figure 1D).

### 3.2 Increased infiltration of macrophages (CD68 ^+^) and leukocytes (CD45 ^+^) was observed in the retina of EAU mice

**FIGURE 2:**
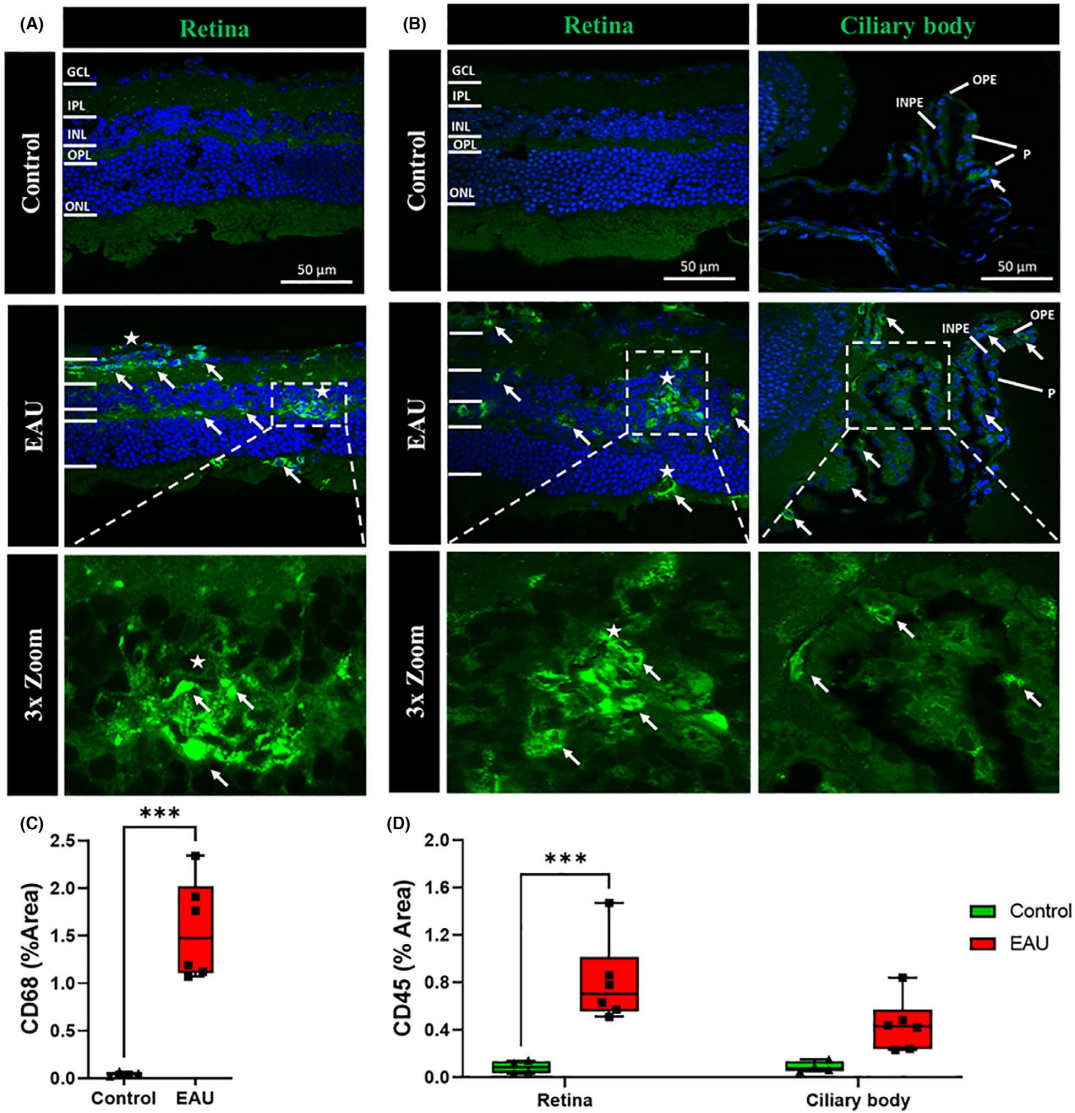
Immunohistochemical images showing inflammatory cells, macrophages (CD68^+^), and leukocytes (CD45^+^) in the retina, ciliary body, and cornea in EAU (experimental autoimmune uveitis) mice (*n* = 6) compared to controls (*n* = 4). (A) Expression of CD68^+^ (green) was upregulated throughout all the layers of the retina (white arrows), with CD68^+^ cells forming clumps in the GCL (ganglion cell layer), IPL (inner plexiform layer), and OPL (outer plexiform layer) (white stars). (B) CD45^+^ (green) expression was upregulated in the GCL, IPL, OPL, and ONL (outer nuclear layer) (white arrows), with cells forming clumps in the INL (inner nuclear layer), OPL, and ONL (white stars) of the retina. CD45^+^ cells were also present in the OPE (outer pigmented epithelium) and INPE (inner nonpigmented epithelium) of the ciliary body (white arrows) but did not form clumps. (C) Significant differences in CD68^+^ expression in the retina between the EAU and control groups were calculated using a multiple *t*‐test. The total area of the retina expressing CD68^+^ cells was significantly higher (***p = 0.0004) in EAU mice compared to controls. (D) Significant differences in CD45^+^ expression in the retina and ciliary body between the EAU and control groups were calculated using two‐way ANOVA (analysis of variance) with Sidak's multiple comparisons post hoc test. EAU mice had a significantly increased percentage area of CD45^+^ expression in the retina (***p = 0.0004) while showing only a slight trend of increase in the ciliary body.

Infiltration of inflammatory cells was assessed by quantifying the expression of CD68 and CD45 in the retina, ciliary body, and cornea (Figure 2). A higher number of CD68^+^ cells were observed in the GCL, IPL, OPL, and ONL of EAU mice (Figure 2A, white arrows), with cells forming clumps in the OPL and GCL (Figure 2A, white stars). Similarly, a higher number of CD45^+^ cells were observed in the retina (Figure 2B, white arrows) and ciliary body (Figure 2B, white arrows) of EAU mice, with several cells appearing in clumps (Figure 2B, white stars) in the GCL, IPL, OPL, and ONL. There was no CD68^+^ expression in the ciliary body and cornea nor CD45^+^ expression in the cornea. Expression of CD68^+^ and CD45^+^ was quantified as the percentage area of positive labeling. Expression of CD68^+^ significantly increased in the retina (1.565% ± 0.211% vs. 0.045% ± 0.01%, *p* = 0.0004) of EAU mice compared to controls (Figure 2C). Significantly higher expression of CD45^+^ was observed in the retina (0.803% ± 0.144% vs. 0.085% ± 0.027%, *p* = 0.0004) of EAU mice compared to controls (Figure 2D). In the ciliary body, CD45^+^ expression showed a trend toward elevation in EAU mice (0.442% ± 0.910% vs. 0.088% ± 0.023%, *p* = 0.0600) compared to controls, but this was not statistically significant (Figure 2D).

### 3.3 Expression of IL‐17A significantly increased in the retina and ciliary body of EAU mice

**FIGURE 3:**
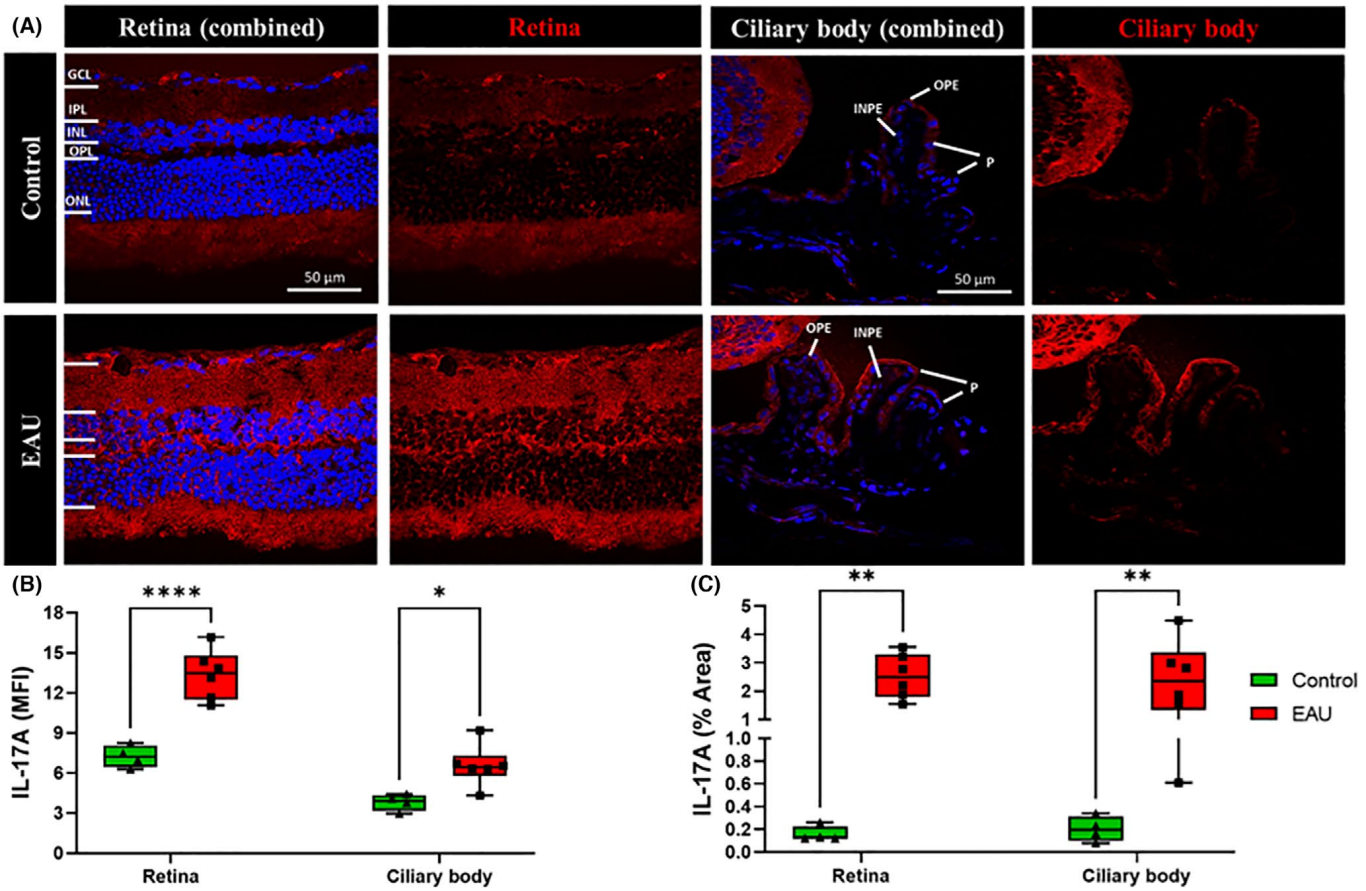
Immunohistochemical images showing the expression of IL‐17A (marker for activated T‐cell subset Th17) in the retina and ciliary body in EAU (experimental autoimmune uveitis) mice (*n* = 6) compared to controls (*n* = 4). (A) IL‐17A (red) expression was upregulated throughout the retina and ciliary body in EAU mice compared to controls. (B) Expression of IL‐17A was measured as MFI (mean fluorescence intensity) throughout the retina and ciliary body, and significant differences between the EAU and control groups were calculated using two‐way ANOVA (analysis of variance) with Sidak's multiple comparisons post hoc test. IL‐17A expression was significantly upregulated in the retina (****p < 0.0001) and ciliary body (*p = 0.0100) of EAU mice. (C) Significant differences between the percentage area covered by IL‐17A labeling in the retina and ciliary body of the EAU and control groups were calculated using two‐way ANOVA with Sidak's multiple comparisons post hoc test. EAU mice had a significantly increased percentage area of IL‐17A expression in the retina (**p = 0.0013) and ciliary body (**p = 0.0020) compared to the respective tissues in control mice.

The expression of IL‐17A, a marker for the activation of the T‐cell subset, Th17, was assessed in the retina, ciliary body, and cornea in EAU and control mice. The expression of IL‐17A increased in the retina and ciliary body in EAU mice compared to controls (Figure 3A), whereas no labeling was observed in the cornea. The upregulation of IL‐17A was quantified as the MFI and percentage area of IL‐17A positive labeling. MFI of IL‐17A labeling significantly increased throughout the retina (3.367 ± 0.762 vs. 7.238 ± 0.416, *p* < 0.0001) and ciliary body (6.563 ± 0.634 vs. 3.815 ± 0.311, *p* = 0.0100) of EAU mice compared to the respective tissues in control mice (Figure 3B). Similarly, the percentage area of IL‐17A positive labeling was significantly upregulated in the retina (2.527% ± 0.321% vs. 0.158% ± 0.034%, *p* = 0.0013) and ciliary body (2.395% ± 0.549% vs. 0.203% ± 0.055%, *p* = 0.0026) of EAU mice compared to the respective tissues in control mice (Figure 3C).

### 3.4 Expression of NLRP3 was significantly elevated in multiple layers of the retina and cornea of EAU mice

**FIGURE 4:**
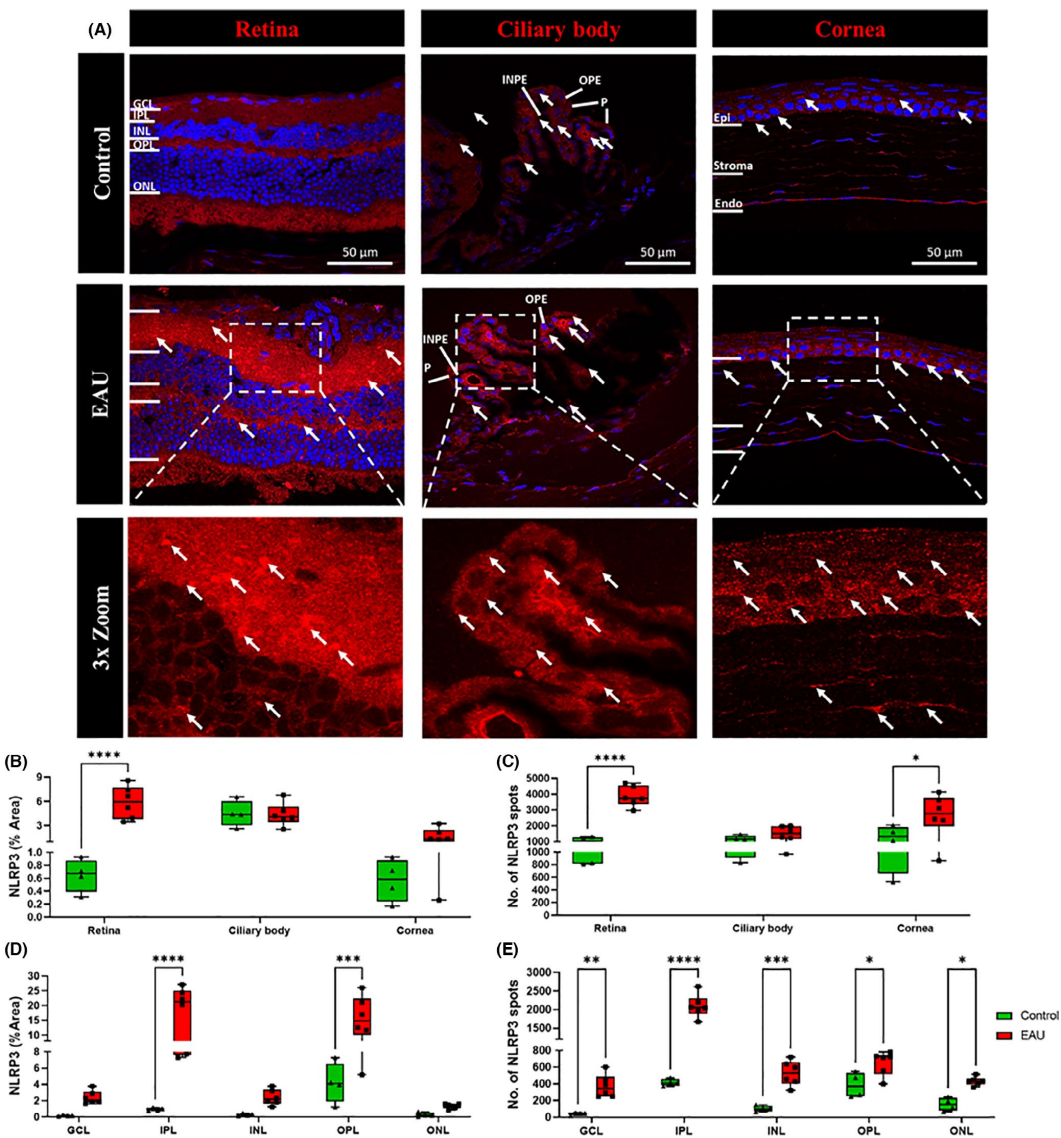
Immunohistochemical images showing upregulation of the inflammasome marker, NLRP3, in the retina, ciliary body, and cornea in EAU (experimental autoimmune uveitis) mice (*n* = 6) compared to controls (*n* = 4). (A) NLRP3 (red) expression was upregulated in the GCL (ganglion cell layer), IPL (inner plexiform layer), OPL (outer plexiform layer), and ONL (outer nuclear layer) (white arrows) and in the corneal epithelium (Epi) and stroma (white arrows) in EAU mice compared to the respective tissues in control mice. There was no difference in NLRP3 expression in the ciliary body between both groups. (B) Significant differences in the percentage NLRP3 area within the retina, ciliary body, and cornea between the EAU and control groups were calculated using two‐way ANOVA (analysis of variance) with Sidak's multiple comparisons post hoc test. EAU mice had a significantly increased percentage area of NLRP3 expression in the retina (****p < 0.0001) but not in the ciliary body or cornea. (C) NLRP3 spot counts were significantly higher in the retina (****p < 0.0001) and cornea (***p = 0.0100) but not in the ciliary body in EAU mice compared to controls. (D) The percentage area of NLRP3 expression was significantly upregulated in the IPL (***p < 0.0001) and OPL (***p = 0.0005) of the retina as calculated using two‐way ANOVA with Sidak's multiple comparisons post hoc test. (E) The number of NLRP3 spots was also significantly upregulated in EAU mice compared to controls in all retinal layers: GCL (**p = 0.0070), IPL (****p < 0.0001), INL (inner nuclear layer) (***p = 0.0004), OPL (*p = 0.0400), and ONL (**p = 0.0300).

Inflammasome activation was assessed by measuring the upregulation of the NLRP3 protein in the retina, ciliary body, and cornea (Figure 4). Results showed an increase in NLRP3 expression in all layers of the retina in EAU mice, with high labeling in the IPL and OPL (Figure 4A, white arrows). NLRP3 expression also increased in the corneal epithelium and stroma of EAU mice compared to controls. Expression in the ciliary body was similar for both EAU and control mice (Figure 4A).

When quantified as total percentage area of NLRP3 positive labeling, a significant increase was observed in the retina (5.875% ± 0.823% vs. 0.645% ± 0.128%, *p* < 0.0001) of EAU mice compared to controls, whereas there was no significant difference in NLRP3 expression in the ciliary body (4.348% ± 0.579% vs. 4.490% ± 0.806%, *p* = 0.9910) or cornea (1.613% ± 0.409% vs. 0.568% ± 0.165%, *p* = 0.5630) (Figure 4B). The percentage area of NLRP3 expression was significantly higher in the IPL (18.160% ± 3.496% vs. 0.913% ± 0.083%, *p* < 0.0001) and OPL (15.608% ± 3.020% vs. 4.163% ± 1.241%, *p* = 0.0005) of EAU mice compared to the respective retinal layers in control mice (Figure 4D). Significantly higher NLRP3 spot counts were observed in the retina (3861.0 ± 264.0 vs. 1040.0 ± 129.0, *p* < 0.0001) and cornea (2749.0 ± 470.0 vs. 1307.0 ± 326.0, *p* = 0.0100) but not ciliary body (1528.0 ± 176.0 vs. 1148 ± 125, *p* = 0.7830) of EAU mice compared to the respective tissues in control mice (Figure 4C). Within retinal layers, the number of NLRP3 spots was found to be increased in the GCL (372.0 ± 56.0 vs. 42.0 ± 7.0, *p* = 0.0070), IPL (2096.0 ± 127.0 vs. 417.0 ± 21.0, *p* < 0.0001), INL (527.0 ± 61.0 vs. 103.0 ± 17.0, *p* = 0.0004), OPL (651.0 ± 59.0 vs. 385.0 ± 74.0, *p* = 0.0400), and ONL (430.0 ± 21.0 vs. 153.0 ± 39.0, *p* = 0.0300) of EAU mice compared to the respective retinal layers in control mice (Figure 4E).

### 3.5 Cleaved caspase 1 was upregulated in the retina, especially the ONL, in EAU mice

**FIGURE 5:**
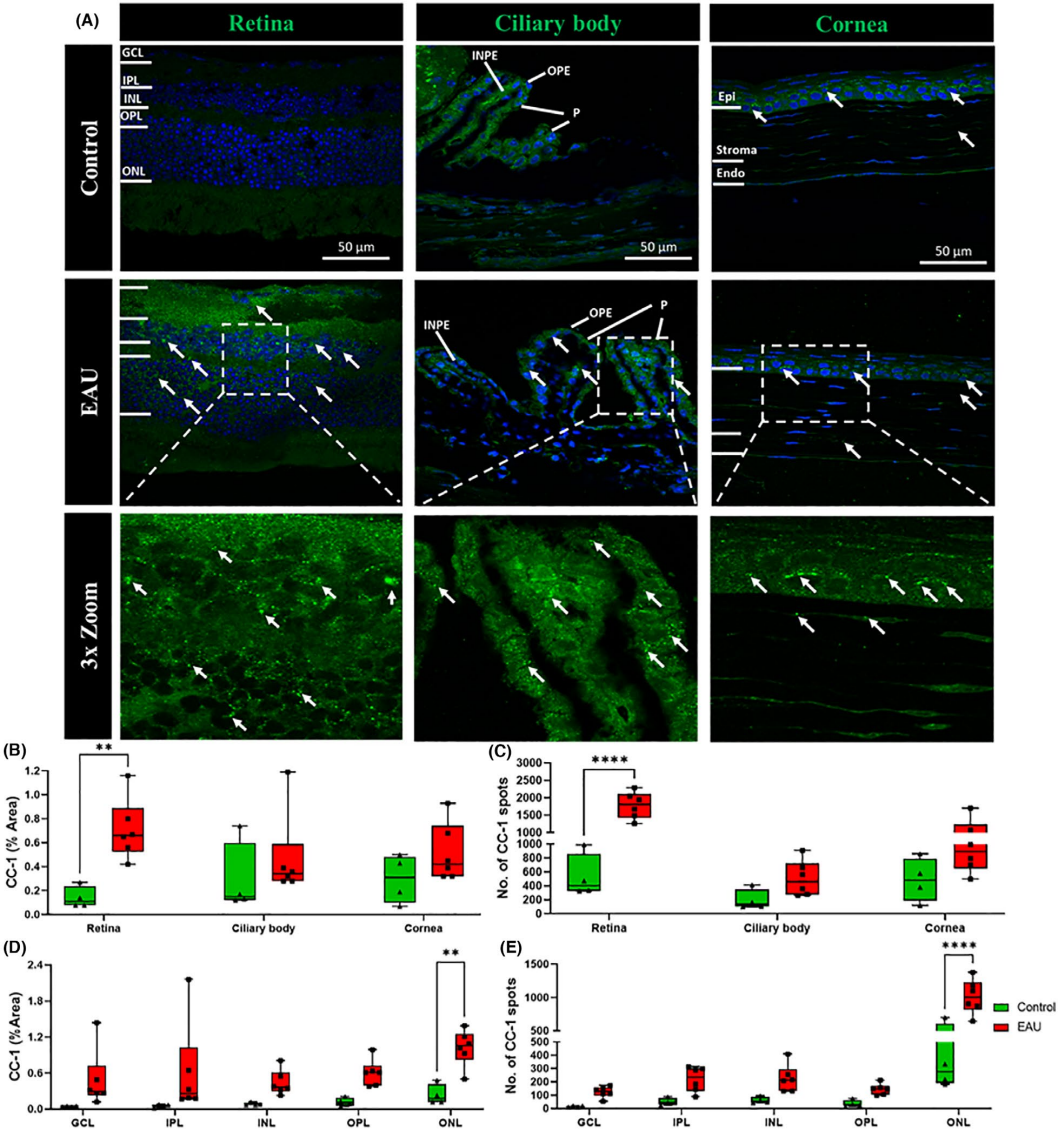
Immunohistochemical images showing upregulation of inflammasome activation marker CC1 (cleaved caspase 1) in the retina, ciliary body, and cornea in EAU (experimental autoimmune uveitis) mice (*n* = 6) compared to controls (*n* = 4). (A) CC1 (green) expression was upregulated in the GCL (ganglion cell layer), IPL (inner plexiform layer), OPL (outer plexiform layer), and ONL (outer nuclear layer) (white arrows) of the retina but not in the ciliary body or cornea of EAU mice compared to controls. (B) Significant differences in the percentage area of CC1 positive labeling in the retina, ciliary body, and cornea between the EAU and control groups were calculated using two‐way ANOVA (analysis of variance) with Sidak's multiple comparisons post hoc test. EAU mice had a significantly increased percentage area of CC1 expression in the retina (**p = 0.0080) but not in the ciliary body or cornea. (C) The CC1 spot count was also significantly higher in the retina (****p < 0.0001) but not in the ciliary body or cornea in EAU mice compared to control mice as determined using two‐way ANOVA with Sidak's multiple comparisons post hoc test. (D) Within retinal layers, the percentage area covered by CC1 labeling was significantly upregulated only in the ONL (**p = 0.0080) as calculated using two‐way ANOVA with Sidak's multiple comparisons post hoc test. (E) The CC1 spot count was upregulated only in the ONL (****p < 0.0001).

Inflammasome activation was also evaluated by quantifying CC1 expression (Figure 5). CC1 expression was upregulated in all retinal layers in EAU mice (Figure 5A, white arrows) compared to control mice. CC1 expression was also observed in various layers of the ciliary body and cornea (Figure 5A, white arrows), with increased expression particularly in the corneal epithelium (Figure 5A, white arrows).

When quantified as percentage area of CC1 positive labeling (Figure 5B), there was a significant increase in the retina (0.710% ± 0.104% vs. 0.143% ± 0.045%, *p* = 0.008) but not in the ciliary body (0.470% ± 0.145% vs. 0.290% ± 0.150%, *p* = 0.6570) or cornea (0.515% ± 0.099% vs. 0.298% ± 0.101%, *p* = 0.5130) of EAU mice compared to the respective tissues in controls. Within retinal layers, CC1 was found to be significantly increased in the ONL (1.023% ± 0.123% vs. 0.238% ± 0.085%, *p* = 0.0080) in EAU mice (Figure 5D). Results were confirmed when individual CC1 spots were counted (Figure 5C). A significantly higher CC1 spot count was found in the retina (1783.0 ± 156.0 vs. 531.0 ± 157.0, *p* < 0.0001) of EAU mice compared to controls. There was a trend toward more CC1 spots in the ciliary body (506.1 ± 104.2 vs. 196.1 ± 73.2, *p* = 0.3980) and cornea (961.0 ± 171.3 vs. 486.4 ± 156.2, *p* = 0.0990) of EAU mice compared to controls, but this was not statistically significant. The CC1 spot count was also shown to be significantly higher in the ONL (1015.0 ± 106.0 vs. 359.2 ± 119.3, *p* < 0.0001) of the retina in EAU mice compared to control mice (Figure 5E).

### 3.6 Expression of connexin43 significantly increased in the retina, ciliary body, and cornea of EAU mice

**FIGURE 6:**
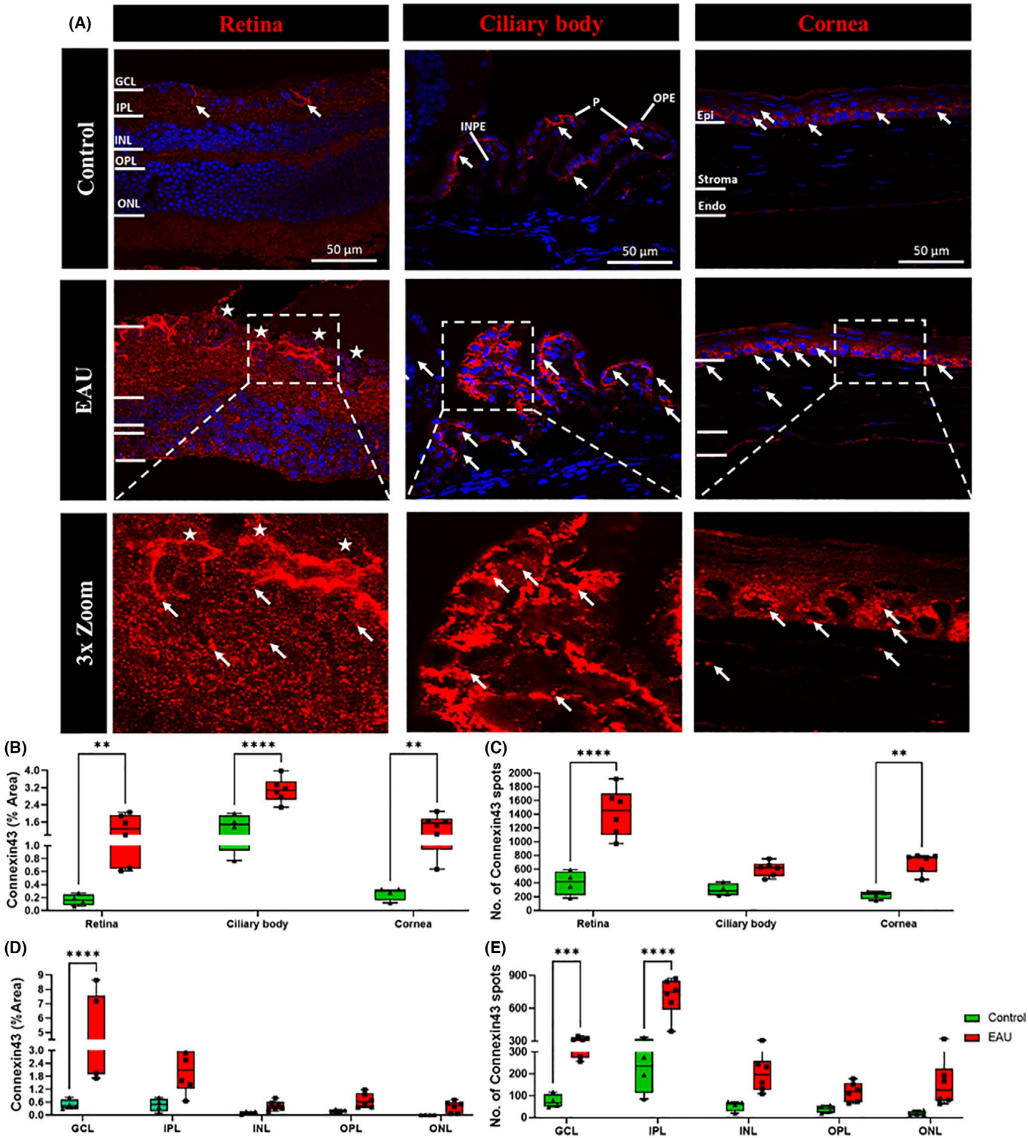
Immunohistochemical images showing upregulation of connexin43 in the retina, ciliary body, and cornea in EAU (experimental autoimmune uveitis) mice (*n* = 6) compared to controls (*n* = 4). (A) Connexin43 (red) expression was upregulated in all layers of the retina (white arrows) and ciliary body and in the corneal epithelium and stroma in EAU mice compared to controls. High expression of connexin43 was also observed around clumps of cells in the GCL (ganglion cell layer) and IPL (inner plexiform layer) of the retina. (B) Significant differences in the connexin43 percentage area in the retina, ciliary body, and cornea between the EAU and control groups were calculated using two‐way ANOVA (analysis of variance) with Sidak's multiple comparisons post hoc test. EAU mice had a significantly increased percentage area of connexin43 expression in the retina (**p = 0.0040), ciliary body (****p < 0.0001), and cornea (**p = 0.0040). (C) The connexin43 spot count was significantly higher in EAU mice in the retina (****p < 0.0001) and cornea (**p = 0.0030) but not in the ciliary body as determined using two‐way ANOVA with Sidak's multiple comparisons post hoc test. (D) Within the retinal layers, the percentage area covered by connexin43 labeling was significantly higher only in the GCL (****p = <0.0001) as calculated using two‐way ANOVA with Sidak's multiple comparisons post hoc test. (E) The connexin43 spot count was also significantly higher in the GCL (***p = 0.0007) and IPL (****p < 0.0001).

Upregulation of connexin43, a potential target protein involved in inflammasome activation, was assessed in the retina, ciliary body, and cornea. Connexin43 was observed to be highly expressed in all layers of the retina and ciliary body and in the epithelium of the cornea in EAU mice (Figure 6A, white arrows). In the retina, high levels of connexin43 labeling were observed around clumps of cells in the GCL extending into the IPL (Figure 6A, white stars). When quantified as percentage area of connexin43 positive labeling (Figure 6B), results showed a significant increase in the retina (1.300% ± 0.256% vs. 0.165% ± 0.043%, *p* = 0.0040), ciliary body (3.087% ± 0.232% vs. 1.445% ± 0.259%, *p* < 0.0001), and cornea (1.42% ± 0.209% vs. 0.265% ± 0.05%, *p* = 0.0040) in EAU mice compared to the respective tissues in the control group. Within retinal layers, connexin43 expression was found to be significantly increased only in the GCL (4.478% ± 1.163% vs. 0.493% ± 0.111%, *p* < 0.0001) of EAU mice (Figure 6D). Results were confirmed when individual connexin43 spots were counted (Figure 6C). A significantly increased number of connexin43 spots were observed in the retina (1429.0 ± 142.2 vs. 403.3 ± 89.4, *p* < 0.0001) and cornea (695.5 ± 58.6 vs. 227.2 ± 28.1, *p* = 0.0030). There was a trend toward an increased number of connexin43 spots in the ciliary body (604.0 ± 43.3 vs. 301.2 ± 45.2, *p* = 0.0600) of EAU mice compared to controls. The connexin43 spot count was also shown to be significantly higher in the GCL (306.6 ± 13.2 vs. 75.6 ± 15.2, *p* = 0.0007) and IPL (709.2 ± 71.3 vs. 223.0 ± 54.1, *p* < 0.0001) of EAU mice compared to controls (Figure 6E).

### 3.7 Retinal expression of IL‐17A correlated positively with NLRP3, CC1, and connexin43 expression in EAU mice

**FIGURE 7:**
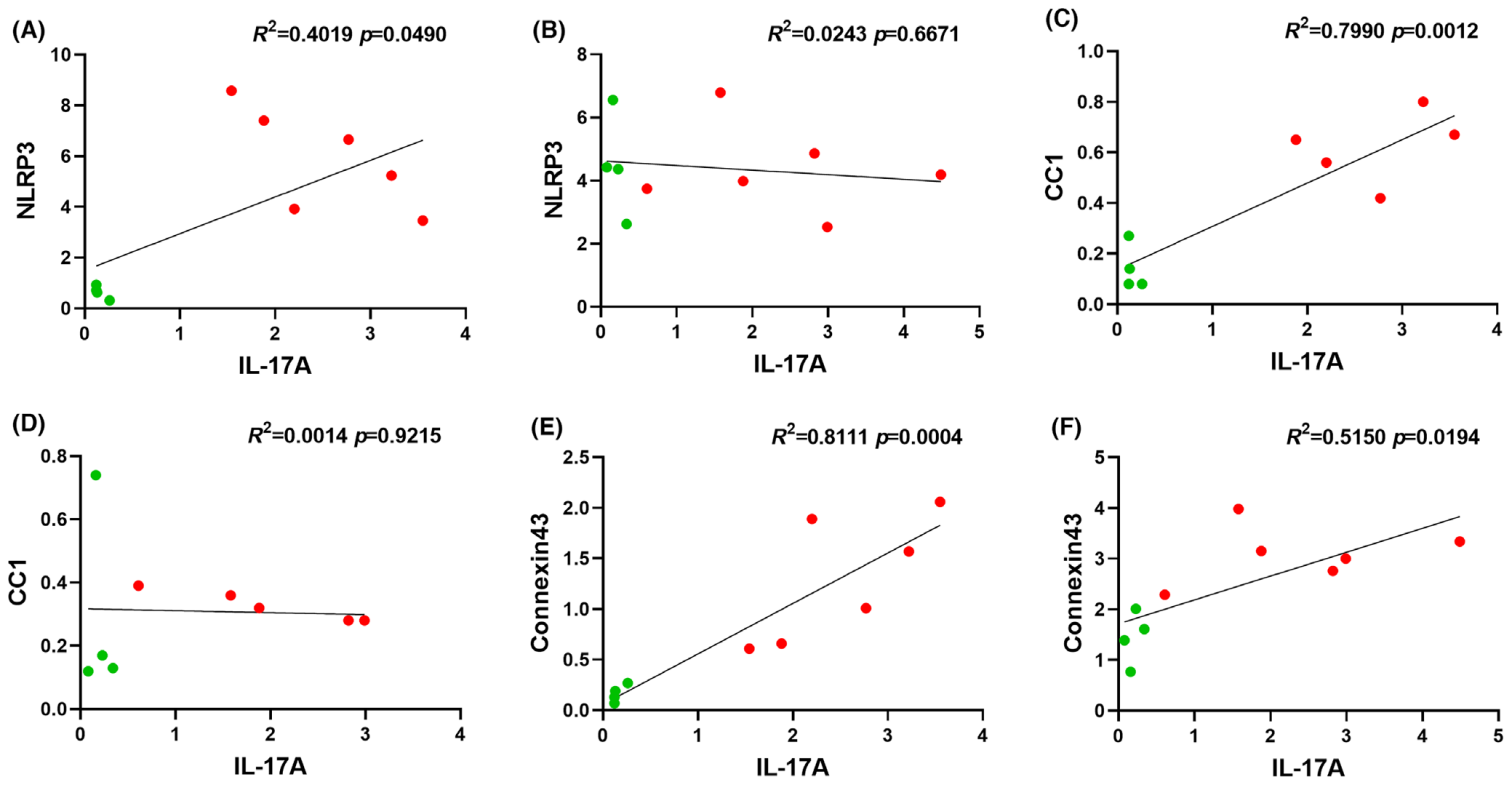
Simple linear regression between IL‐17A and NLRP3 (nucleotide‐binding oligomerization domain and leucine‐rich repeat receptor‐3), CC1 (cleaved caspase 1), and connexin43 in the retina and ciliary body of EAU (experimental autoimmune uveitis) (*n* = 6, red dots) and control (*n* = 4, green dots) mice. (A, C, E) IL‐17A exhibited a positive linear correlation with the expression of NLRP3, CC1, and connexin43 in the retina of EAU mice. (B, D, F) IL‐17A exhibited a positive linear correlation with the expression of connexin43 but not NLRP3 or CC1 in the ciliary body of EAU mice.

A correlation between the percentage area of IL‐17A and NLRP3, CC1, and connexin43 expression was evaluated using simple linear regression (Figure 7). Results showed that the expression of IL‐17A correlated significantly with the expression of NLRP3 (Figure 7A; *R* ^2^ = 0.4019, *p* = 0.0490), CC1 (Figure 7C; *R* ^2^ = 0.7990, *p* = 0.0012), and connexin43 (Figure 7E; *R* ^2^ = 0.8111, *p* = 0.0004) in the retina of EAU mice. In the ciliary body, on the contrary, IL‐17A did not correlate with NLRP3 (Figure 7B; *R* ^2^ = 0.0243, *p* = 0.6671) and CC1 (Figure 7D; *R* ^2^ = 0.001, *p* = 0.9215) expression but exhibited a significant correlation with connexin43 labeling (Figure 7F; *R* ^2^ = 0.515, *p* = 0.0194). Because there was no IL‐17A expression in the cornea, a correlation could not be made with respect to NLRP3, CC1, and connexin43 expression in this tissue.

## 4 DISCUSSION

Previous studies have highlighted that infectious uveitis is generally driven by the innate immune system, whereas the adaptive immune system, especially Th cells, plays a leading role in the manifestation of noninfectious uveitis.^7^, ^18^, ^28^ Although this might be true in most cases, recent studies have shown that activation of innate immune pathways, such as inflammasome, is associated with the development of noninfectious uveitis.^19^, ^29^ It is important to note that most of these studies either focus on downstream IL‐1β production alone^30^ or only track EAU progression for up to 6 weeks when active inflammation is thought to be “spontaneously resolved”.^19^, ^25^ In contrast, there are studies that highlight inflammasome‐independent regulation of IL‐1β^31^ and continuation of active inflammation, especially in the vitreous and retinal layers, beyond 6 weeks in the EAU mouse model.^26^ Shome et al.^26^ showed that active inflammation was present on the retina up to 12 weeks post EAU induction. Therefore, the present study investigated the correlation between inflammatory (immune cells) and inflammasome activation (immune and nonimmune cells) markers in eyes collected 12 weeks post EAU induction. Our results clearly show, even after 12 weeks, that immune cells, both resident (e.g., microglia) and infiltrating peripheral (e.g., Th17 cells), are active throughout the eye, and their activation directly correlates to NLRP3 inflammasome activation in immune and nonimmune cells.

Initially, activation and expression of microglia and astrocytes/Müller cells in the eye were investigated (Figure 1). Microglia, specialized macrophages present in the central nervous system (CNS), have been shown to play a major role in EAU^32^ by facilitating the infiltration of leukocytes into the eye.^33^ Microglia can be found in the retina, but a recent study reported that circulating myeloid cells, including microglia, are also present in the ciliary body and cornea of transgenic and choroidal neovascularization mouse models.^34^, ^35^ In line with these studies, we observed a significant increase in microglia numbers within the retina, ciliary body, and cornea of EAU mice compared to control animals (Figure 1A,B). These data indicate that EAU increases microglial proliferation, circulation, and infiltration throughout the eye, not just in the retina. We also observed several microglial cells forming clumps around circular structures within the GCL (Figure 1A, white stars), which have previously been identified as granulomas and have been shown to be associated with sight‐threatening complications such as retinal atrophy and scarring.^36^

The contribution of immune cells to the inflammatory environment in EAU was further supported by the increased number of macrophages (CD68^+^ cells) and leukocytes (CD45^+^ cells) found within the retina and ciliary body (Figure 2A,B, white arrows). These results suggest a loss of BRB integrity that could contribute to infiltration of immune cells from the periphery into the posterior segment of the eye. Interestingly, CD68 and CD45 labeling was particularly concentrated around granulomas (Figure 2A,B, white stars), which supports the idea that they comprise infiltrating immune cells.^26^ IL‐17A, secreted by Th17 cells that are important for the development of autoimmune uveitis,^6^, ^7^, ^9^ was also elevated in the retina and ciliary body in EAU mice. Immune cell infiltration into the vitreous (vitritis) and retina (retinitis) has previously been confirmed in EAU mice using fundus and OCT imaging as well as hematoxylin and eosin staining.^25^, ^26^, ^37^, ^38^ However, these studies concluded between 4 and 6 weeks and were unable to highlight the extent of inflammatory damage in retinal layers caused by the infiltrating cells. In contrast, our findings clearly show that even after 12 weeks, resident and peripheral immune cells, including microglia, leukocytes, macrophages, and Th17 cells, are clearly active in the posterior (retina) and intermediate (ciliary body) segments of the eye, thus highlighting the chronic nature of inflammation in the EAU model.

In addition to the infiltrating immune cells, we investigated the activity of astrocytic and Müller cells (GFAP^+^ cells) that have been associated with certain progressive ocular inflammatory conditions such as diabetic retinopathy.^22^ We observed the upregulation of GFAP expression in all the retinal layers, especially around granulomas (Figure 1D, white stars). Astrocytes are antigen‐presenting cells^39^ that play a major role in the pathogenesis of ocular inflammation, especially by recruiting peripheral immune cells into the retina.^40^ When activated, astrocytes produce increased amounts of GFAP (Figure 1A), indicating the occurrence of astrocytosis that occurs during severe ocular inflammation.^40^ Astrocytosis has been observed in other diseases where NLRP3 has been implicated in the development of ocular inflammation observed in models of diabetic retinopathy and dry AMD.^41^, ^42^, ^43^ Müller cells act as modulators of inflammatory responses within the retina, producing pro‐inflammatory cytokines such as IL‐6 that are important for the differentiation of Th17 cells.^44^ The activation of Müller cells has been associated with NLRP3 inflammasome pathway activation.^45^ In sum, in addition to astrocytosis, Müller cell activation in EAU mice highlights an inflammatory microenvironment in the retina and supports the involvement of NLRP3 inflammasome activation.

To further understand the pathway involved in the activation of resident and recruitment of circulating immune cells in the EAU, we investigated NLRP3 inflammasome activation in the retina, ciliary body, and cornea. We observed significant NLRP3 upregulation in the retina and cornea as well as significantly increased CC1 expression in the retina (Figure 4). We also observed that NLRP3 levels were increased throughout the retina, with a significant elevation in the IPL and OPL (Figure 4D). Conversely, CC1, which is responsible for the maturation of IL‐1β and IL‐18,^46^ was elevated only in the ONL (Figure 5D). Perhaps, therefore, although NLRP3 may be primed within all retinal layers, activation and subsequent autolytic cleavage of caspase 1 may occur only in the ONL. On the contrary, the difference in localization between NLRP3 and CC1 could also be attributable to the possible migration of cleaved CC1 to different regions, including the nuclear layers of the retina.^41^, ^47^, ^48^ This could explain the lack of CC1 expression in the ciliary body or cornea if the cleaved CC1 is migrating only toward the inner layers of the retina. Another possible explanation is that although the inflammasome is being primed (increased NLRP3) in various regions of the eye (retina and cornea), it is only being activated (increased CC1) in infiltrating immune cells, which are primarily present in the retina. However, further investigation is needed to validate this hypothesis in the EAU mouse model. Overall, our study clearly indicates that, even after 12 weeks, the NLRP3 inflammasome is not only primed but also activated in nonimmune and immune cells in the retina, leading to “sterile inflammation” within the eye, starting at the posterior and spreading to the anterior region.^13^, ^49^ Furthermore, the strong correlation between IL‐17A expression and NLRP3 as well as CC1 in the retina of EAU mice further emphasizes a connection between inflammasome pathway and adaptive immune system in noninfectious uveitis. Based on our data, inhibiting inflammasome activation and pro‐inflammatory cytokine maturation could be crucial to treat ocular inflammation in models such as EAU.

Finally, to investigate the potential of targeting the inflammasome pathway in EAU, we assessed connexin43 protein expression. Connexin43 hemichannels are upstream of the inflammasome pathway and have been shown to mediate ATP release from nonimmune and resident immune cells.^50^, ^51^ ATP release, in turn, acts as an inflammasome activation signal.^52^ Connexin43 hemichannel blockade has previously been efficacious in various ocular and CNS inflammatory diseases.^22^, ^42^, ^53^, ^54^ Our results showed that the connexin43 protein was upregulated in the retina, ciliary body, and cornea of EAU mice compared to controls (Figure 6). High expression was observed not only around resident nonimmune cells (Figure 6A, white arrows) but also around granulomas (Figure 6A, white stars). Similar to NLRP3 and CC1, we also found increased connexin43 expression to correlate with the upregulation of IL‐17A in the retina. Although we do not have direct evidence of connexin43 hemichannel opening and connexin43 hemichannel‐mediated inflammasome activation by release of the ATP priming signal, this may play a crucial role in Th17 infiltration/proliferation. However, further study is required to substantiate this. A limitation of the present study was the inability to investigate the expression of undocked connexin43 hemichannels given that available antibodies recognize both hemichannels and gap junctions, especially because undocked hemichannels specifically are responsible for the pathogenesis of ocular inflammatory conditions.^50^, ^55^, ^56^

In conclusion, we have shown an increased expression of resident (Iba‐1 and GFAP) and peripheral (CD45, CD68, and IL‐17A) immune cell markers in conjunction with the activation of the NLRP3 inflammasome (NLRP3 and CC1) and a possible upstream activation signal (via connexin43 hemichannel opening). We demonstrated inflammasome activation in resident nonimmune and circulating immune cells, and our results confirm our previous findings;^26^ that is, there is still active inflammation in the EAU model after 12 weeks post induction and the activated inflammasome contributes to the pathophysiology and chronicity of noninfectious uveitis. Research in the field of treatment of noninfectious uveitis so far has largely been focused on the action of immune cells in response to inflammatory or autoimmune triggers although ignoring the capacity of nonimmune cells to contribute to this response. This is where the study of inflammasomes becomes important. The inflammasome machinery is present not only in innate immune cells (leukocytes, neutrophils, and monocytes) but also in resident cells (endothelial cells, epithelial cells, retinal pigment epithelium (RPE)) of the “immune privileged” eye.

As such, our data support the idea of targeting the inflammasome pathway, which is upstream in the inflammatory cascade and could be an efficacious and efficient treatment, thus reducing the number of adjuvant therapies required for treating complex chronic autoimmune conditions such as noninfectious uveitis.

## AUTHOR CONTRIBUTIONS

**Avik Shome:** Conceptualization; data curation; formal analysis; investigation; methodology; validation; writing – original draft. **Ilva D. Rupenthal:** Conceptualization; formal analysis; funding acquisition; project administration; resources; software; supervision; validation; writing – review and editing. **Rachael L. Niederer:** Conceptualization; data curation; formal analysis; investigation; project administration; software; supervision; writing – review and editing. **Odunayo O. Mugisho:** Conceptualization; data curation; formal analysis; funding acquisition; methodology; project administration; supervision; validation; writing – review and editing.

## FUNDING INFORMATION

This research was funded by a Maurice and Phyllis Paykel Trust Grant (203134). Avik Shome was supported by a Buchanan Ocular Therapeutics Unit Doctoral Scholarship. Odunayo O. Mugisho is supported by a Neurological Foundation First Postdoctoral Research Fellowship (2001 FFE), an Auckland Medical Research Foundation Grant (1121013), and an Auckland Medical Research Foundation Postdoctoral Fellowship (1323001). Ilva D. Rupenthal's directorship was supported by the Buchanan Charitable Foundation, with part of her salary also supported by the Health Research Council of New Zealand (20/317).

## CONFLICT OF INTEREST STATEMENT

Odunayo O. Mugisho is an inventor on patents relating to inflammasome pathway regulation in chronic diseases.

## ETHICS APPROVAL

All procedures were carried out in accordance with local legislations approved by the University of Auckland Animal Ethics Committee (AEC2882 approved on 9 November 2020) and in accordance with the Association for Research in Vision and Ophthalmology (ARVO) resolution and the ARRIVE guidelines for use of animals in research.

## Data Availability

All data generated or analyzed during this study are included in this published article.
